# A novel live-dead staining methodology to study malaria parasite viability

**DOI:** 10.1186/1475-2875-12-190

**Published:** 2013-06-07

**Authors:** Erica M Pasini, Denise van den Ierssel, Henri J Vial, Clemens HM Kocken

**Affiliations:** 1 Biomedical Primate Research Centre, Lange Kleiweg 161, 2288, GJ Rijswijk, The Netherlands; 2 Dynamique des Interactions Membranaires Normales et Pathologiques, CNRS UMR 5235, Université Montpellier 2, Place Eugene Bataillon, 34095 Montpellier, Cedex 05, France

## Abstract

**Background:**

Malaria is a major health and socio-economical problem in tropical and sub-tropical areas of the world. Several methodologies have been used to assess parasite viability during the adaption of field strains to culture or the assessment of drug potential, but these are in general not able to provide an accurate real-time assessment of whether parasites are alive or dead.

**Methods:**

Different commercial dyes and kits were assessed for their potential to allow for the real-time detection of whether a blood stage malaria parasite is dead or alive.

**Results:**

Here, a methodology is presented based on the potential-sensitive mitochondrial probe JC-1, which allows for the real-time visualization of live (red staining) and/or dead (absence of red staining) blood stage parasites *in vitro* and *ex vivo*. This method is applicable across malaria parasite species and strains and allows to visualize all parasite blood stages including gametocytes. Further, this methodology has been assessed also for use in drug sensitivity testing.

**Conclusions:**

The JC-1 staining approach is a versatile methodology that can be used to assess parasite viability during the adaptation of field samples to culture and during drug treatment. It was found to hold promise in the assessment of drugs expected to lead to delayed death phenotypes and it currently being evaluated as a method for the assessment of parasite viability during the adaptation of patient-derived *Plasmodium vivax* to long-term *in vitro* culture.

## Background

Malaria, a disease caused by apicomplexan parasites of the genus *Plasmodium*, with *Plasmodium falciparum* causing 250–500 million clinical cases and up to 1.2 million deaths every year, is a major health- and socio-economical problem in the tropical and sub-tropical areas of the world [1]. Today, malaria occurs in 90 countries: an estimated 1.26 billion people (29% of the world’s population) live in areas where malaria is currently on the rise, while 400 million people live with endemic malaria unchanged by current control measures [2]. While vector and transmission control measures (e.g. bed nets) are in place in most of the risk countries [3,4] and the world’s scientific community is looking for effective vaccines [5], drugs are the weapon of choice both for malaria prophylaxis [6,7] and treatment [8,9].

As the focus shifted towards malaria eradication, there is a need to adapt new field strains and additional parasite species. For example, *Plasmodium vivax*, the second most important human malaria, has been largely neglected due to absence of *in vitro* culture systems and there is an increasing need to adapt it to long-term *in vitro* culture for research purposes and develop new therapies. In addition, there are challenges derived from the rapidly spreading resistance to existing anti-malarial drugs, mainly in *P*. *falciparum* and from the need to tackle emerging zoonoses (e.g. *Plasmodium knowlesi*[10,11]). Thus, the search for potent anti-malarials with innovative mechanism of action remains a priority.

The challenges inherent to the adaptation of field strains to culture, to the development of currently lacking, innovative, continuous parasite culture methods for specific malaria parasite species (e.g. *P*. *vivax* or the closely related primate malaria *Plasmodium cynomolgi* and to *in vivo*, *ex vivo* and *in vitro* drug potency studies would benefit from the availability of a sensitive and specific live-dead staining of parasites that would make it possible to asses real time parasite viability.

A number of *ex vivo* and *in vitro* reference methods have been established for the screening of anti-malarial activity, whereby drug potency is usually expressed as the concentration required to inhibit parasite growth by 50% (IC_50_). The Mark III WHO Giemsa thick blood smear method [12] is the gold standard in the field, while a variety of medium- to high-throughput drug screening methods based on either nucleic acid incorporation of radio-labelled hypoxanthine [13], SYBR green I dye [14,15], the detection of parasite specific enzymes (HRP-2 [16,17], LDH [18-20]) or the use of transgenic fluorescent parasites [21] have been introduced for laboratory use. Other screening methods are pathway specific and have been used to determine whether a compound can interfere with a certain specific parasite metabolic pathway (e.g. haemozoin formation [20,22]). Drawbacks of these methods include, but are not limited to, the subjective nature of the measurement, its reproducibility, the use of radioactive material, and the need for dedicated spaces, specialized equipment and specific disposal procedures.

Here, a methodology is presented based on the potential-sensitive mitochondrial probe JC-1, which allows for the real-time visualization of live (red staining) and/or dead (absence of red staining) parasites *in vitro* and *ex vivo*. This method is applicable across parasite species and strains (*P*. *falciparum* FCR3 and NF54, *P*. *knowlesi H*, *P*. *cynomolgi M*) and allows visualizing all parasite blood stages including gametocytes. This methodology has been assessed also for use in drug sensitivity testing and it is currently being evaluated as a method for the assessment of parasite viability during the adaptation of patient-derived *P*. *vivax* to long-term *in vitro* culture.

## Methods

### Parasites

*Plasmodium falciparum* NF54, FCR3 strains and *P*. *knowlesi* H strain were cultured *in vitro* in human A + red blood cells (RBC) (*P*. *falciparum*) (Sanquin Blood Bank, The Netherlands) or in rhesus monkey RBC (*P*. *knowlesi*) in complete medium composed of RPMI-1640 (with 25 mM HEPES, Invitrogen) supplemented with gentamycin and 10%-20% human serum (A+) in standard gassed (5% O_2_, 5% CO_2_, 90% N_2_) culture flasks and kept under shaking in an incubator at 37°C [23-25]. Parasites for live/dead staining were obtained at a parasitaemia between 3-10%, stained with JC-1 as reported below and immediately evaluated with a fluorescence microscope (Nikon Microphot-FXA equipped with green filter XF22, blue filter UV, red/green XF53, red XF40 and a Nikon FX35 PX camera).

*Plasmodium cynomolgi ex vivo* samples were obtained from infected rhesus monkeys from unrelated experiments after ethical permission from the ethical committee as required under Dutch law. Blood containing *P*. *cynomolgi* M strain trophozoite and gametocyte stages at around 2% parasitaemia was collected, immediately stained with JC-1 (using the protocol reported below) and the staining was evaluated as described above.

### Parasite staining with JC-1 and drug assays using LDH or JC-1 staining as read-out

Drug assays were performed in culture flasks or 96-well plates by adding serial drug dilutions to synchronized *P*. *falciparum* or *P*. *knowlesi* ring-stage cultures as previously described [13,26,27]. Briefly, the concentration range in each of the JC-1 assays was based on the IC_50_ values, previously obtained using pLDH and hypoxanthine radioactive testing for each drug on the specific parasite strain. From stock solutions of each drug, 10-fold serial dilutions were made and plated in triplicate. As a read out of drug activity either the established pLDH enzymatic assay [18,19] or JC-1 staining were used.

For JC-1 staining for fluorescent microscope-based counting and parasite visualization, a 200 μl sample was taken from a standard culture or *ex vivo* experiment, spun down at 930 × g for 10 min at RT and medium was removed. When drug testing was carried out in plates, the plate was spun down and the medium removed. Pellets containing parasite infected erythrocytes were washed five times with sterile PBS at RT and re-suspend at 5% haematocrit and 1% parasitaemia in 500 μl (plate: 100 μl) Ringer’s solution either containing 4 nM DAPI (for manual counting of slides, Nikon microphot-FXA microscope) or 10 μl/ml Hoechst, Bisbenzimide H 33258 (Sigma, for counting with a high-content imager, the Operetta®, Perkin Elmer). Five μl JC-1 dye (Molecular Probes, Invitrogen) was added to the sample (final concentration: 2 μM), which was then incubated for 30 minutes at 37°C in the dark with continuous agitation. After the incubation period, cells were washed again 5 times with PBS to remove excess dye. Samples were either used to make wet slides (manual counting) or resuspended in 1 mL PBS and aliquoted as specified below for Operetta based counting.

In infected RBC containing both living and dead parasite, the JC-1 staining is characterized by a diffused green fluorescence (JC-1 monomer) across the RBC and parasite’s cytoplasm. Living parasites are further characterized by strong red mitochondrial fluorescence as the JC-1 monomer aggregates in presence of the mitochondrial membrane potential characteristic of a living parasite. This red signal is absent in dead parasites, which have lost their mitochondrial functionality and thus also their mitochondrial membrane potential.

Manual counting was performed using the fluorescence microscope. Up to 50 parasites/slide were counted. If fewer than 50 parasites were seen in slides from drug-treated samples, we assumed the remainder had died and lysed, and they were, therefore, noted as dead.

Operetta® based counting was performed as follows: after plated parasites were JC-1 stained and re-suspended in 1 ml PBS as described above, 50 μl of the suspension was transferred to a 96-well plate and an additional 50 μl PBS was added. The positive control containing untreated parasites was plated twice, each in triplicate, once at the start of the plate and once at the end to ensure consistency. RBCs were allowed to settle for about 5 min and a snapshot of the wells was taken using the bright-field filter to check for the cell density in each well. If the cells could be viewed individually the density was considered appropriate for fluorescence measurement, otherwise the RBC suspension was adjusted appropriately. In order to achieve accurate counting, pictures of 25 fields per well were taken on which all red (JC-1 aggregate) and green (JC-1 monomer only) parasites were afterwards counted manually. Manual counting was preferred, as Operetta counts of non-adherent cells would require development and validation of new algorithms, which were beyond the scope of our study. Operetta settings used for making the pictures were selected as follows: 40× long Working Distance objective, excitation 50%, transmission 50%. Visualization of the different dyes was performed using the standard Operetta® filters for DAPI, Texas Red and JC-1 pH8 monomer (green). Both gamma and contrast settings were optimized to achieve optimal pictures for assessing the status of the parasites. The intensity values used to name a parasite alive, dying or dead are <350 units dead, >350 < 450 units dying and >450 units alive. These units were validated by conducting side-by-side experiments using the red channel of a fluorescent microscope to evaluate the staining’s correlation with parasite viability: signal absent (parasite dead), faint red signal at times difficult to detect (parasite dying), signal bright red (parasite alive).

All parasites in each well were counted and their state (living, dead, dying) assessed. The untreated controls were used to calculate the final percent inhibition using the formula below. If there were fewer parasites present in the treated well as compared to the mean untreated control, the missing parasites were assessed as dead.

$$\%\;\mathrm{inhibition}=100-\left[\frac{\left(\text{Number living parasites}/\text{total number positive control}\right)\times 100}{\%\;\text{living parasites in positive control}}\right]\times 100$$

## Results and discussion

### JC1 is a viability stain distinguishing living from dead malaria parasites

This study aimed at developing a fluorescent parasite staining protocol that would allow to distinguish live from dead parasites during the adaptation to long-term *in vitro* culture of Plasmodium species, and would also be useful in drug-efficacy assays. For this, we evaluated dyes with differential membrane permeability and/or specifically labelling parasite organelles key to life. Nuclear stains tested in *P*. *falciparum* and *P*. *knowlesi* included SYTO-16, a combination of SYTO-10 and ethidium homodimer-2 and combinations of potential insensitive anionic and cationic dyes (acridine orange with ethidium bromide or propidium iodide), while mitochondrial stains tested in *P*. *falciparum* and *P*. *knowlesi* included MitoTrack, TMRE and the potential-sensitive Mitoprobe JC-1.

Nuclear dyes (e.g. *SYTO*-*16*) used to distinguish live from apoptotic/necrotic eukaryotic cells have different permeability characteristics (neutral or ionic) and staining thus depends on the integrity of the plasma membrane. However, when these dyes are applied to *P*. *falciparum*- and *P*. *knowlesi*-infected RBCs, mature schizonts are systematically stained as dead, due to the increased membrane permeability of the late Plasmodium blood life-cycle stages. Although staining selectivity was observed with dyes of the SYTO family in the case of rings, trophozoites and free merozoites (Additional file 1 and Additional file 2), these dyes were not considered useful for our applications.

**Figure 1:**
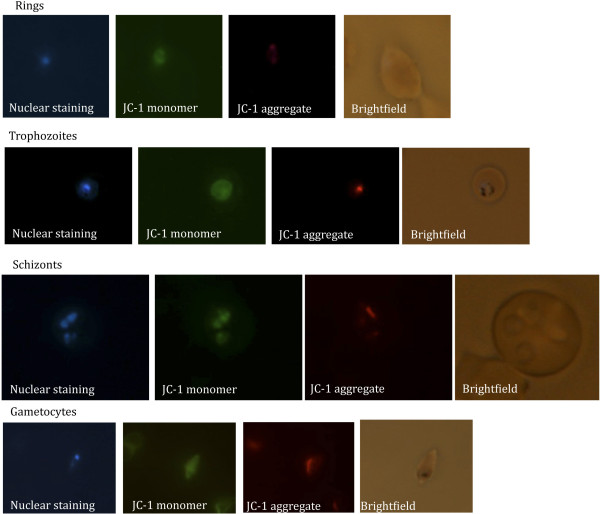
**Living *P. falciparum *parasite stages stained with JC-1. ***Plasmodium falciparum* blood stages (rings, trophozoites, schizonts and gametocytes) stained with JC-1. The first panel in each row shows the staining of the parasite’s nucleus with Hoechst dye (350 nm); the second panel shows JC-1 monomer visible at 488 nm (green channel), which colours the parasite’s cytoplasm; the third panel shows the JC-1 aggregate visible at 568 nm (red channel) staining the parasite’s active mitochondrion; while the last panel shows the corresponding bright field image.

**Figure 2:**
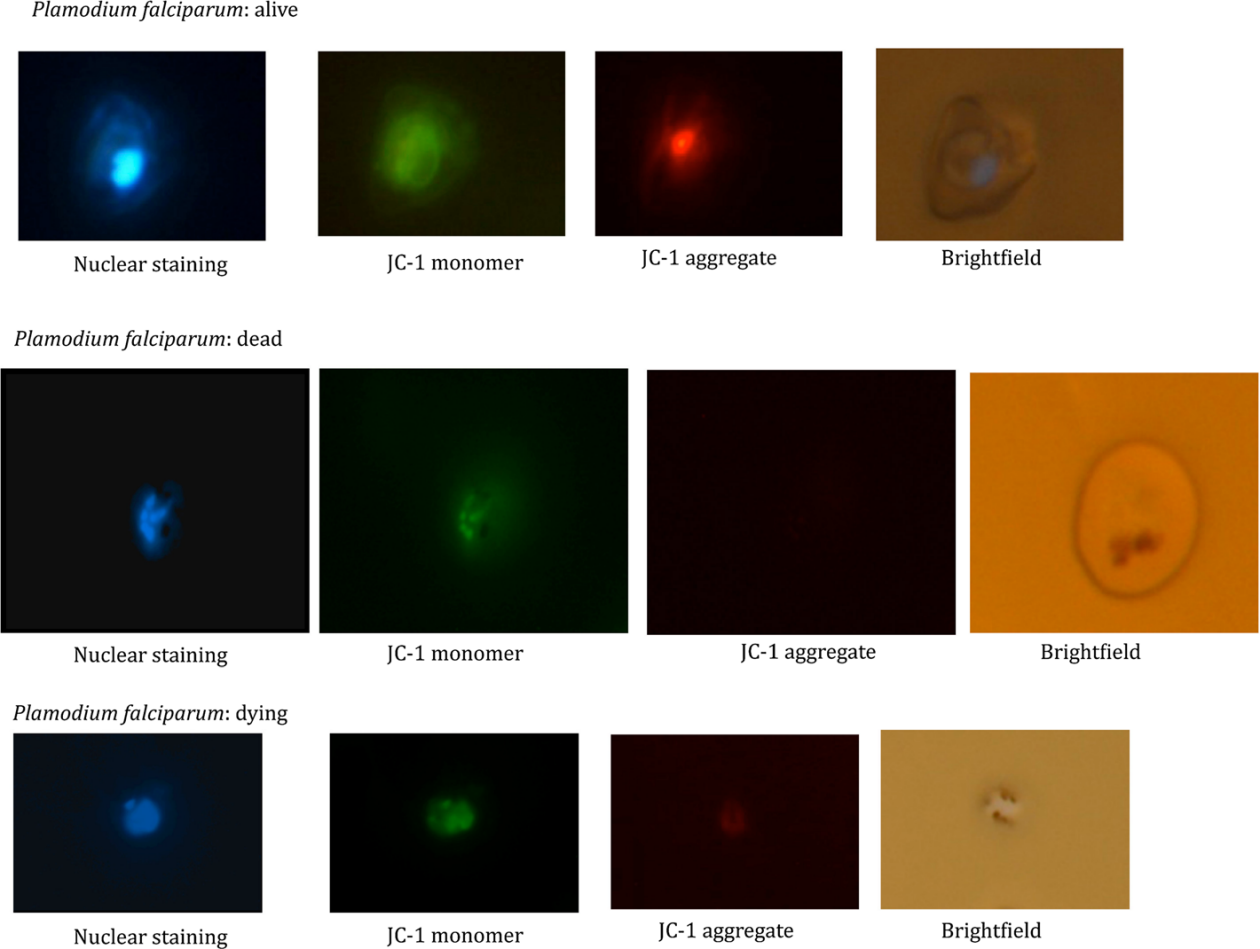
**Living, dead and dying *P. falciparum *schizonts stained with JC-1.** JC-1 staining of *Plasmodium falciparum* schizonts. The first row of images shows a living parasite as can be appreciated by the bright red signal at 568 nm indicating a functioning mitochondrion; the second row of images shows a dead parasite killed using anti-malarial drugs (no signal at 568 nm). In the third series only a faint red signal (sometimes difficult to detect) can be seen at 568 nm indicating that the mitochondrion membrane potential is disrupted and the parasite is dying.

In 1998, Borel *et al*. [28,29] described a new methodology employing a combination of an anionic and a cationic dye (acridine orange/ethidium bromide or bisbenzimide (Hoechst 33258)/propidium iodide) to distinguish living from dead *Toxoplasma gondii*. While acridine orange has been evaluated in the field as an alternative to Giemsa for malaria diagnosis [29-31] and found to be promising, the double dye method did not yield consistent results when *P*. *falciparum* or *P*. *knowlesi* drug-treated blood stage cultures were examined for the number of living and dead parasites. This may be due to the fact that this method is also partially based on differential membrane permeability, as the Hoechst, Bisbenzimide H 33258 (Sigma) dye is able to reach the nucleus when the cell membrane is intact by passive diffusion, while propidium iodide only reaches the nucleus when the membrane is compromised. This yields the same problem for mature schizonts as discussed above. Further, some mitochondrial stains (TMRE, MitoTrack) were evaluated on *P*. *falciparum*- and *P*. *knowlesi*-infected blood cultures, which gave a bright red labelling of living parasites, but gave at times residual vacuolar staining in dead parasites. Another mitochondrial stain, the potential-sensitive Mitoprobe JC-1, responds to the difference in mitochondrial membrane potential in living and dead cells. When the mitochondrial potential is normal (living parasite) the dye aggregates in the mitochondrion, labelling the mitochondrion red; when the potential is disrupted less of the dye aggregates resulting in a faint red signal (dying parasite), which may at times be difficult to detect, while when the potential is absent (dead parasite) the dye is only present in its monomeric form in the cytoplasm, thus labelling the parasite cytoplasm green. The fluorescent JC-1 staining of *in vitro* cultured living *P*. *falciparum* parasites growing in human RBC was evaluated in different parasite stages across the blood stage life cycle (rings, trophozoites, schizonts, merozoites and gametocytes, Figure 1). This demonstrated that all blood stage parasites could be clearly distinguished. Importantly, also mature schizont stages are stained as live parasites, unlike the other staining procedures mentioned above as JC-1 staining is not dependent on parasite membrane permeability, but only on the mitochondrial membrane potential, which is not altered in schizonts. In living *P*. *falciparum* parasites, the JC-1 red staining pattern corresponds to a previously reported pattern in which the malarial mitochondrion was labelled with Discosoma red fluorescent protein (DsRed) [32] or yellow fluorescent protein (YFP) [33]. JC-1 staining of a *P*. *falciparum* culture containing both living and dead parasites demonstrated that living, dying and dead parasites can be easily distinguished (Figure 2), confirming that this staining method is suitable for our purpose.

### Optimization of the JC-1 staining protocol

Next we evaluated different JC-1 staining conditions: wet, live versus dry, fixed staining, extensive versus limited washing before and after staining, different wash media (Ringers, RPMI, PBS) and different incubation times. The most optimal conditions found, measured at 5% haematocrit and 1% parasitaemia to be: i) wet slides of standard cultures (reduces the bleaching); ii) extensive washing with PBS to get rid of all serum residues, as serum interferes with the staining procedure; iii) incubation of the washed parasitized RBCs for 30 minutes at 37°C in the presence of JC-1 dye at a final concentration of 2 μM and iv) extensive washing with PBS after staining to remove excess JC-1 thereby reducing the background. For the final microscopical analysis, cells need to be re-suspended in PBS as Ringers interferes with the detection. The preparations need to be studied immediately after staining to prevent parasites in PBS dying over time or the staining fading with time and/or during prolonged exposure to fluorescent light sources. In general, the nuclear stain allows to quickly recognize parasitized RBCs; the presence of a diffuse green signal defines cytoplasmic staining of any parasite and the additional presence of a bright red signal identifies an intact mitochondrion and thus a viable parasite. The exclusive presence of a cytoplasmic green signal in the absence of a red signal identifies a dead parasite devoid of mitochondrial potential (Figure 2).

Initially, in parallel staining of drug-treated cultures and cultures left purposefully outside the incubator overnight with both Giemsa and JC-1 were carried out, where Giemsa was used as a Gold standard (GS). In brief, 2000 parasites per slide were counted each time and classified either as live or dead. Table 1 shows representative results of repeated, parallel slide counting. As is apparent, slightly more living parasites are estimated in Giemsa slides, than is actually the case when JC-1 is used as a read out. This is probably due to the fact that it is difficult to distinguish between live and dead in early parasite stages stained with Giemsa.

**Table 1:** Giemsa versus JC-1 read-out

**Read-out**	**Amount of dead parasites**	**Amount of live parasites**
Gold Standard = Giemsa	947	1053
JC-1	981	1019

### JC-1 staining is applicable to a variety of plasmodium species

**Figure 3:**
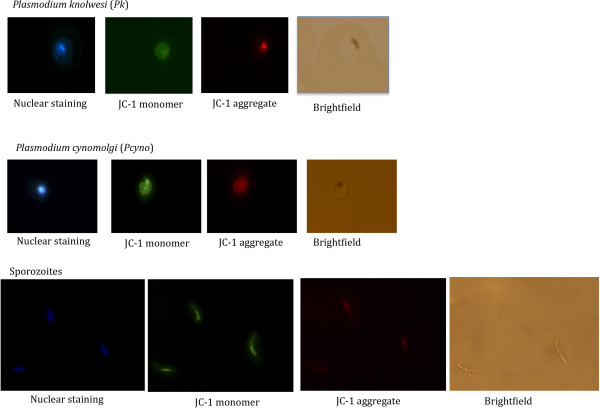
**Living *Plamodium knowlesi *and *P. cynomolgi*stages stained with JC-1.** JC-1 staining of additional Plasmodium *spp* and stages. Top row: *Plasmodium knowlesi in vitro* cultured blood stage trophozoite. Middle row: *Ex vivo* harvested *Plasmodium cynomolgi* blood stage schizont. Bottom row: *P. cynomolgi* salivary gland sporozoite.

After establishing that different life cycle stages of *P*. *falciparum* could be successfully labelled as living, dying or dead using the JC-1 dye, the fluorescent JC-1 staining methodology was extended to *in vitro* or *ex vivo* cultured parasites infecting different types of RBC. Figure 3 shows the staining of *in vitro* cultured *P*. *knowlesi* (non-human primate and human malaria that has been adapted to *in vitro* growth in rhesus normocytes, and *ex vivo P*. *cynomolgi* (a *P*. *vivax*-type parasite infecting non-human primate reticulocytes obtained from rhesus macaque infections. In all cases JC-1 gave a sensitive and specific red signal that could be clearly distinguished from the nuclear staining signal. Living parasites were characterized by a defined red fluorescent structure; while a only diffused green signal was observed in dead parasites (Additional file 3). This demonstrates that the JC-1 staining procedure can be used for different *Plasmodium* species, infecting reticulocytes or normocytes and cultured *in vitro* or obtained from an *in vivo* infection.

The method can thus be considered widely applicable to malaria parasite species and different developmental stages and is therefore a useful tool for quickly monitoring culture viability during the adaptation of new field strains to *in vitro* growth. In particular, we are now developing this methodology further for the assessment of parasite viability during the adaptation of patient-derived *P*. *vivax* to long-term *in vitro* culture. Furthermore, the ability to also visualize sporozoites (Figure 3, a freshly isolated *P*. *cynomolgi* sporozoite) opens the possibility to quickly assess viability e.g. following different cryo-preservation protocols, before testing subsequent hepatocyte invasion potential.

### JC-1 staining can be used as read out in drug assays

**Figure 4:**
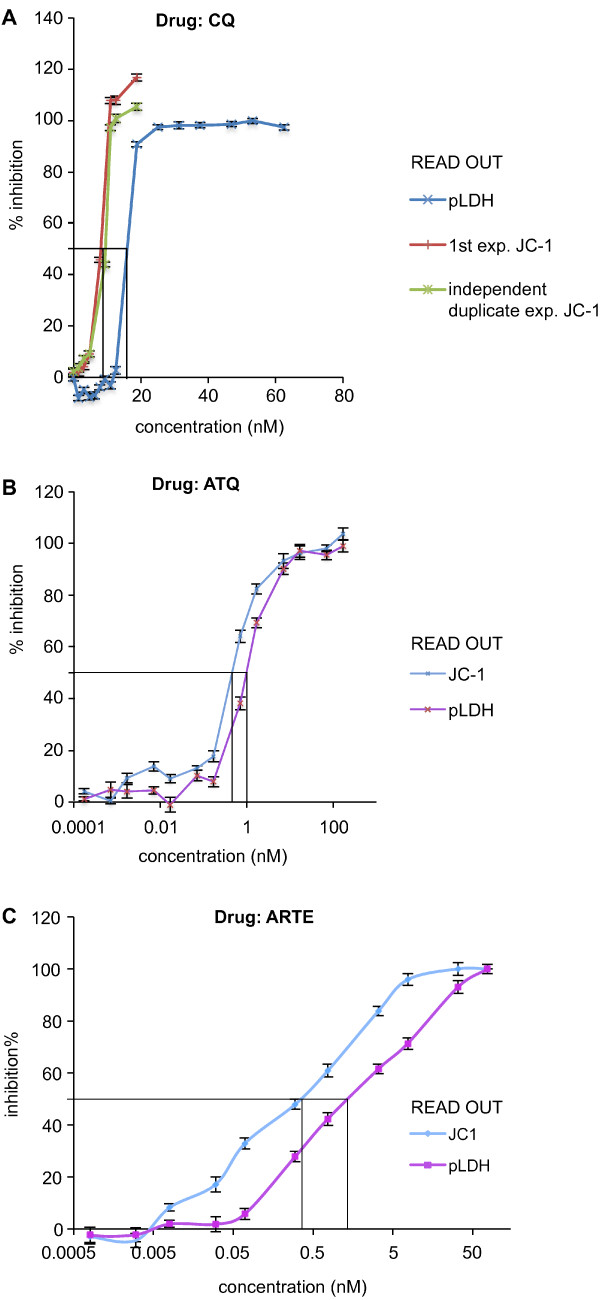
**IC**_**50 **_**determination using JC-1 and/or pLDH in parallel on drug treated*P. falciparum*cultures.** Culture *P. falciparum*-infected erythrocyte were incubated for 48 h in the presence of the indicated drug concentration at haematocrit of 5% and 1% parasitaemia. *Panel****A*** - shows that the inter-experiment variability between 2 separate JC-1 determinations is low (red and violet lines) and that the methodology is more accurate (IC_50_ is consistently lower) than the IC_50_ determination obtained with the pLDH methodology; *Panel****B*** - shows typical curves obtained by splitting parasites derived from a same mother culture into plates, in which they were cultured in parallel under the same environmental conditions in the presence of rising artemisinin concentrations and IC_50_ curves where either determined using the pLDH (violet line) or the JC-1 (blue line) methodology; *Panel****C*** – shows typical curves obtained by splitting parasites derived from a same mother culture into plates, in which they were cultured in parallel under the same environmental conditions in the presence of rising atovaquone concentrations and IC_50_ curves where either determined using the pLDH (violet line) or the JC-1 (blue line) methodology.

As a second objective, the usefulness of the JC-1 staining as a read out to assess parasite viability in drug-treated cultures was evaluated using different prototype drugs i.e. choloroquine (CQ, acting on the food vacuole [34]), atovaquone (ATQ, acting on mitochondria [35]) and artemisinin (ARTE, likely acting through a free radical-based oxidant stress [36,37]). In addition we evaluated a compound series, which targets the parasite’s phospholipid synthesis pathways (G25 and albitiazolium) [38-42] and is currently being developed for clinical use. Both manual counting of non-fixed slides from drug-treated parasite cultures using a fluorescence microscope and manual counting of real-time pictures (25 fields per well) taken from plated, drug-treated parasite cultures by an automatic high-content imaging system (Operetta®) were used to monitor drug action. Parasite sensitivities to any of the above mentioned drugs either on *P*. *falciparum* or *P*. *knowlesi* was measured using JC-1 as a read-out with both counting methodologies and were found to yield the typical sigmoid curve indicating a linear relationship between drug concentration and parasite inhibition between the threshold dose level and the plateau dose level over a limited number of drug concentrations. Although the developed methodology is reproducible and the inter-experiment reproducibility is relatively high (Figure 4 Panel A), the manual counting of living non-fixed parasite slides using a fluorescent microscope had an important limitation. Due to the long-time needed to count each slide (3 h for a 24 well plate drug assessment), the non-treated control wells counted at the beginning and end of the procedure gave different results: up to 3-5% more dead parasites were found in the control slides counted at the end of the experiment versus the ones counted in the beginning. This is due to the fact that live parasites do not like to be kept in wet slide conditions for too long. The use of Operetta® pictures of the wells and manual counting of parasites in those pictures solved this problem: comparison of control wells at the beginning and end in each plate showed high (98-99%) consistency, due to the fact that the parasites’ condition was fixed by the pictures, which were taken in rapid succession across the whole plate and could thus not deteriorate over time. The imager procedure appears more sensitive than the fluorescent microscope counting at the low (IC_20_) drug concentrations and more accurate at high drug concentrations (IC_90_) when there are only very few living parasites left as many more fields are photographed in rapid succession per well and counted in comparison to manually counted slides in which a time-field counting balance needs to be found as the amount of dead parasites in control wells increases over time as explained above.

Parallel experiments using the LDH and JC-1 staining methodologies were carried out using CQ, ATQ (Figure 4 Panel B) and ARTE (Figure 4 Panel C) treated *P*. *falciparum FCR3* and *P*. *knowlesi* cultures. Experiments were performed in triplicate for each drug concentration. With all three drugs, the JC-1 staining methodology appears to be more accurate as the JC-1 read out is a direct measure of parasite viability obtained by staining the whole parasite population (including rings) and not by measuring a surrogate parasite marker produced only by the trophozoite and schizont stages (pLDH or LDH enzyme activity). Repeated JC-1 staining experiments over once cycle (48 h for *P*. *falciparum* and 24 h for P. *knowlesi*) have on average yielded IC_50_s for the *P*. *falciparum FCR3* and *P*. *knowlesi H* strains treated with the different drugs, which are lower than with the LDH methodology (Tables 2 and 3). These experiments were repeated several times and it appears that the gain in sensitivity over a longer life-cycle (*P*. *falciparum*: 48 h) is in general higher than over a shorter life-cycle (*P*. *knowlesi*: 24 h). This reflects the fact that despite the lethal effect exerted by the drugs, the production of the malaria-specific pLDH protein is not stopped rapidly and the measurement of the pLDH protein, which is still being synthesized by the parasite thus biases this type of test. However, the JC1 staining reflects the mitochondrial status of the parasite at the time when the test is applied.

**Table 2:** **IC**_**50 **_**ranges from in parallel experiments**

**Drug**	CQ in nM*		ATQ in nM*		ARTE in nM*	
**Assay**	***LDH***	***JC-1***	***LDH***	***JC-1***	***LDH***	***JC-1***
**Pf FCR3**	14.1-16.6	8.8-10.9	0.7-1.3	0.5-0.9	0.1-0.9	0.03-0.1
**Pk H**	9.38-10.9	7.8-9.7	2-2.6	2.1-2.7	1.1-1.6	0.8-1.2

*All IC_50_ ranges were determined on an average of 8–10 experiments where JC-1 and LDH experiments were carried out side by side.

**Table 3:** **IC**_**50 **_**average with standard deviation**

Drug in nM*	**Assay**	**Pf FCR3**	**Pk H**
CQ	*LDH*	15.71 ± 1.23	10.4 ± 1.03
	*JC-1*	10.33 ± 0.97	8.3 ± 0.92
ATQ	*LDH*	1.16 ± 1.41	2.54 ± 0.86
	*JC-1*	0.78 ± 0.72	2.27 ± 0.31
ARTE	*LDH*	0.52 ± 0.83	1.53 ± 0.46
	*JC-1*	0.07 ± −0.59	0.95 ± 0.72

*Mean IC_50_s determined on an average of 8–10 experiments where JC-1 and LDH experiments were carried out side by side.

**Figure 5:**
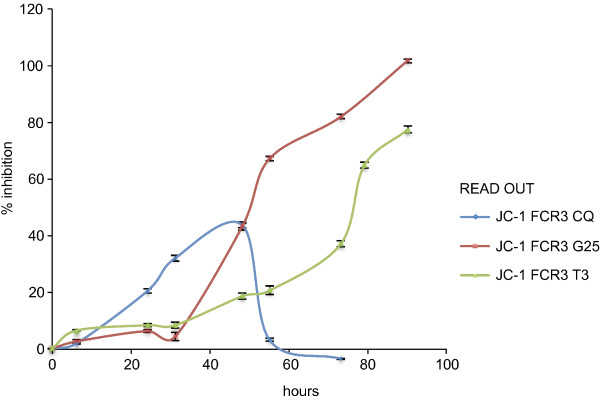
**Delayed death phenotypes monitored using JC-1 on synchronized *P. falciparum FCR3 *cultures exposed to chloroquine and the phospholipid pathway inhibitors prototype drug. ***P. falciparum FCR3* culture (5% hematocrit and 0.1 starting parasitaemia) were exposed to 7.82 nM chloroquine and the phospholipid pathway inhibitors prototype drugs (2.25 nM albitiazolium and 0.6 nM G25). After two hours of contact with the drugs, cell were washed and resuspended in drug-free fresh complete medium. JC-1 staining was then used to monitor parasite viability over the course of at least 3 cycles to detect any delayed drug effects. The results are expressed as means ± SEM (n = 3). Exposure of a ring culture to the IC_50_ of chloroquine for 2 h has no delayed effect but acts on trophozoites and schizonts during the first cycle as expected (blue line), but exposure of the same ring culture to the IC_50_ of G25 and albitiazolium for only 2 hours results in a potent delayed death effect, which for G25 reaches 100% within the second parasite cycle (red line) and for albitiazolium probably within beyond the third (green line) cycle.

Choline analogues G25 and albitiazolium constitute a new class of anti-malarial drugs that target the malarial membrane biogenesis through the phospholipid metabolism. The clinical candidate albitiazolium (formerly named T3 and SAR97276) [42] is currently evaluated in clinical phase II trials to treat severe malaria by the parenteral route. Choline analogues give unreliable IC_50_ results, when assayed with the traditional LDH test [43]. It has been observed that *P*. *falciparum* left in the presence of G25 or albitiazolium [41,43,44] for a few hours incurred in parasite disappearance in the following life cycle. Compound potency is associated to their properties of accumulating in a non-reversible way within the intra-erythrocytic parasites, which irremediably impaired parasite viability while the parasite morphology is affected more belatedly. This indicates that the drug operates through a Trojan horse effect and its real pharmacological effect is dissociated from the effects on parasite morphology and metabolism [41,43]. Therefore, the methodology was further tested to establish whether it would be appropriate to assess these delayed parasite collapses, by exposing both *P*. *falciparum* FCR3 (Figure 5) and *P*. *knowlesi* H cultures to G25 and albitiazolium at their IC_50_ for 2 hours using CQ as a reference) [43] and followed the parasitaemia development into the next cycle using JC-1 as a read-out (Figure 3). The IC_50_s were first determined using radio-labelled hypoxanthine [13] after exposure to the drugs over 1 cycle, 48 h for *P*. *falciparum* and 24 h for *P*. *knowlesi*). IC_50_ for *P*. *knowlesi* were 0.36 mM (albitiazolium), 1.38 mM (G25) and 5.62 nM (CQ) respectively; while the IC_50_s for *P*. *falciparum* were 2.25 nM (albitiazolium), 0.6 nM (G25) and 7.82 nM (CQ) respectively. When parasite were incubated with the drugs at their IC50 for only 2 h followed by the washing out of the drugs, a potent delayed effect of G25 on *P*. *falciparum* FCR3 (Figure 5, red line) was found, since the full effect of the drug (100% inhibition calculated with the formula reported above for each time point) is observed in the second cycle (past the 24 h). Albitiazolium seems to have a similar effect, but with a delayed collapse even into the third cycle (Figure 5, green line), while as expected CQ (Figure 5, blue line) has no delayed-death effect and acts within the first cycle on trophozoites and schizonts. Results obtained with *P*. *knowlesi* H (Additional file 4) also indicate that after incubation of the parasite with the choline analogues at their IC50 for only 2 h, both G25 and albithiazolium exerted delayed pharmacological effects that are observed during the cycle following the contact with the drug. Altogether, results support the conclusion indicating that use of the JCI staining may be a suitable alternative method to LDH for assessing the IC_50_ of compounds suspected of having delayed death effects.

## Conclusions

The JC-1 staining approach is a versatile methodology that can be used to assess parasite viability during the adaptation of field samples to culture and during drug treatment. On-going efforts aimed at establishing *P*. *vivax* blood stage cultures will particularly benefit from the use of the described method. In particular, this methodology appears to be more accurate and reliable than Giemsa staining of fixed parasites, which rarely gives clear quantitative evidence of whether a parasite culture is in good shape or not.

In the context of the assessment of a compound’s anti-malarial potency, this methodology offers reproducible, robust results comparable to established methods such as the LDH IC_50_ test, while being in general more accurate. Furthermore, a live stain such as JC-1 offers the possibility to follow compounds overtime, thus opening the way to the assessment of delayed death drug phenotype.

## Abbreviations

CQ: Chloroquine; ARTE: Artemisinin; ATQ: Atovaquone; IC50: Half maximal inhibitory concentration; pLDH: Plasmodium lactate dehydrogenase.

## Competing interests

The authors declare that they have no competing interests.

## Authors’ contributions

EP and CK designed the research; DvdI and EP performed the research; HV contributed reagents; EP and CK analysed the data and wrote the manuscript. All authors read and approved the final manuscript.

## Supplementary Material

Additional file 1 ***P*. *falciparum * and *P*. *knowlesi *parasites stained with the mitochondrial dyes TMRE and Mitotrack.** The mitochondrial dye TMRE and Mitortrack appear to give rise to a very aspecific, diffused signal when compared to a well defined signal of the potential-sensitive mitochondrial dye JC-1 (Figures 1, 2, 3). Both TMRE and Mitotrack appear to stain vacuoles and the cytoplasm of the parasites. This raises the question on whether they are indeed labelling specifically the parasites’ mitochondria. Click here for file

Additional file 2 **Living *P*. *falciparum *and *P*. *knowlesi *parasites stained with the nuclear Syto-16 dye and co-stained with the mitochondrial Mitotrack dye.** In eukaryotic cells, Syto-16 is used to distinguish live (no stain) from apoptotic /necrotic cells (nuclei stained green). The dye is able to cross the plasma membrane of the cell only if it is compromised and thus enter the cell nucleus staining it green. However, as *Plasmodium* schizonts due to their increased membrane permeability, the Syto-16 dyes labels living schizonts as dead by staining their nuclei green. Click here for file

Additional file 3 **Dead *P*. *knowlesi *parasites stained with JC-1.** JC-1 staining of drug-treated *Plasmodium knowlesi* culture showing a dead parasite as can be appreciated by the absence of signal at 568 nm. Click here for file

Additional file 4 **Delayed death phenotypes monitored using JC-1 on synchronized *P*. *knowelsi H *cultures exposed to chloroquine and the phospholipid pathway inhibitors prototype drug.***P*. *knowlesi H* culture (5% hematocrit and 0.1 starting parasitaemia) were exposed to 5.62 nM chloroquine and the phospholipid pathway inhibitors prototype drugs (1.38 nM albitiazolium and 0.36 nM G25). After two hours of contact with the drugs, cell were washed and resuspended in drug-free fresh complete medium. JC-1 staining was then used to monitor parasite viability over the course of at least 3 cycles to detect any delayed drug effects. The results are expressed as means ± SEM (n = 3). Exposure of a ring culture to the IC_50_ of chloroquine for 2 h has no delayed effect but acts on trophozoites and schizonts during the first cycle as expected (violet line), but exposure of the same ring culture to the IC_50_ of G25 and albitiazolium for only 2 hours results in a potent delayed death effect, which for G25 reaches 100% within the second parasite cycle (blue line) and for albitiazolium probably within beyond the third (red line) cycle. Click here for file
