# Supplementary figures and images for: A novel live-dead staining methodology to study malaria parasite viability
Source: Malar J. 2013 Jun 7;12:190. doi: 10.1186/1475-2875-12-190 (PMC3680332; doi:10.1186/1475-2875-12-190)

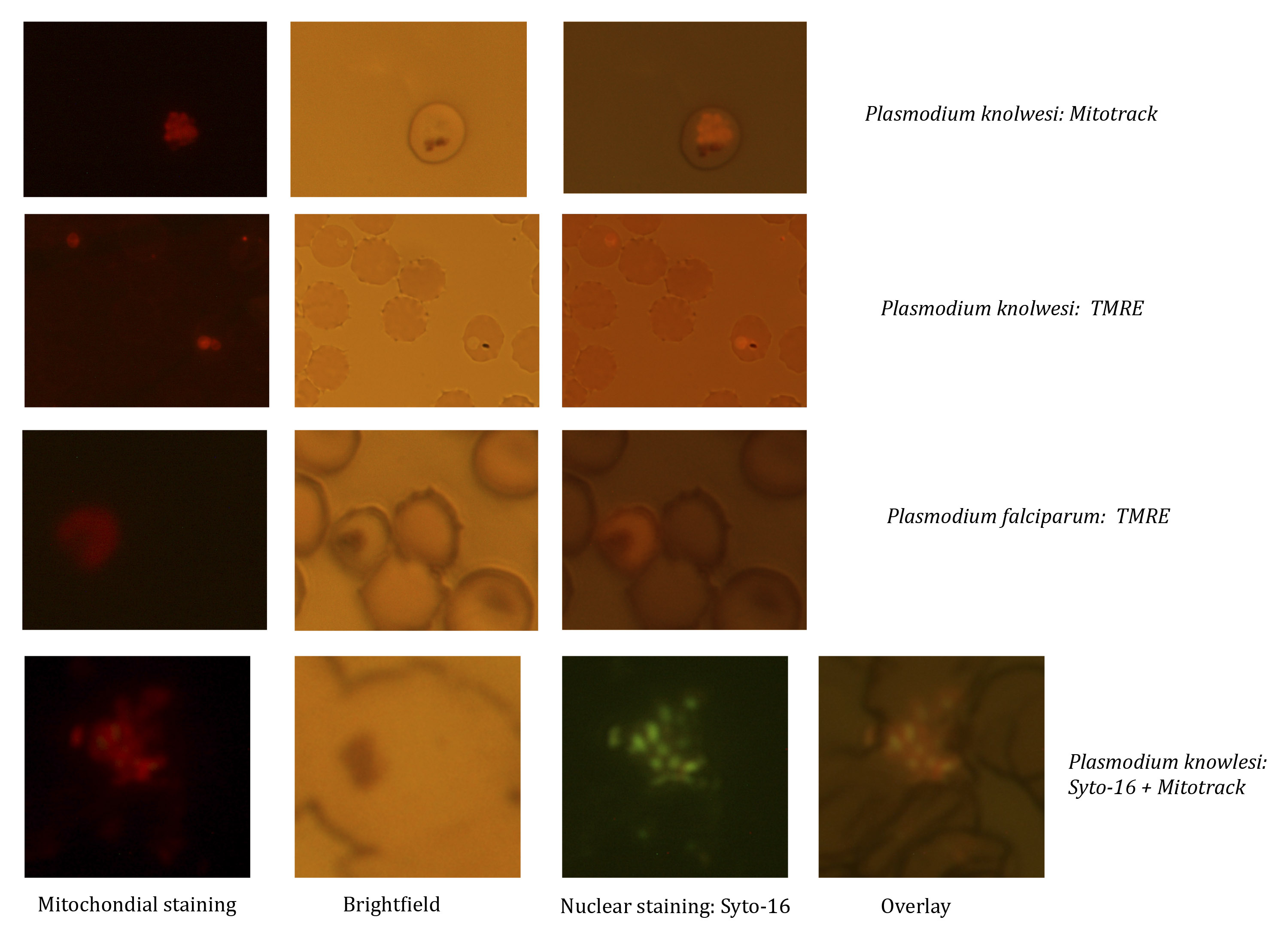

Supplement: Additional file 1 — P. falciparum and P. knowlesi parasites stained with the mitochondrial dyes TMRE and Mitotrack. The mitochondrial dye TMRE and Mitortrack appear to give rise to a very aspecific, diffused signal when compared to a well defined signal of the potential-sensitive mitochondrial dye JC-1 (Figures 1, 2, 3). Both TMRE and Mitotrack appear to stain vacuoles and the cytoplasm of the parasites. This raises the question on whether they are indeed labelling specifically the parasites’ mitochondria. [file 1475-2875-12-190-S1.jpeg]

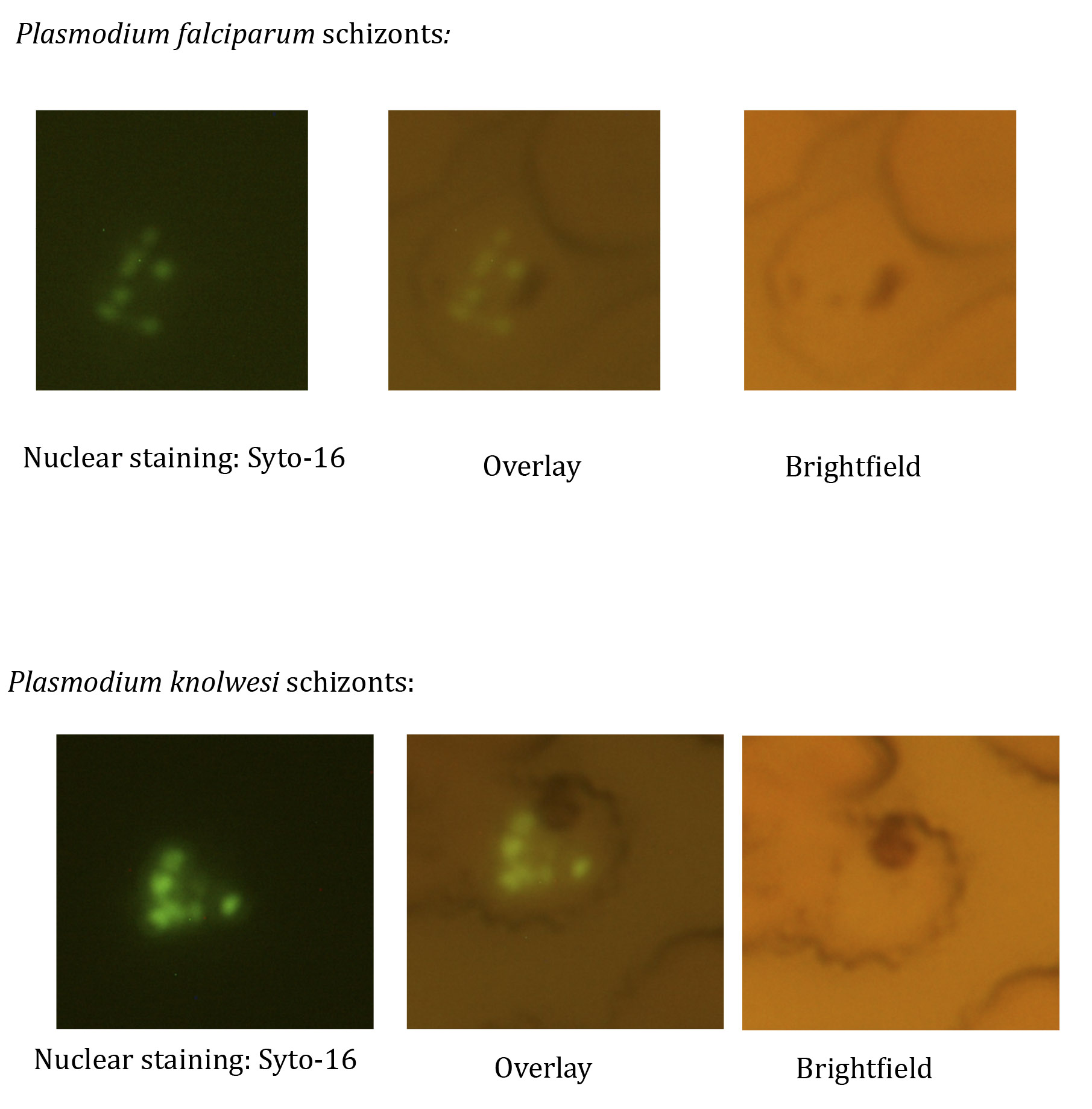

Supplement: Additional file 2 — Living P. falciparum and P. knowlesi parasites stained with the nuclear Syto-16 dye and co-stained with the mitochondrial Mitotrack dye. In eukaryotic cells, Syto-16 is used to distinguish live (no stain) from apoptotic /necrotic cells (nuclei stained green). The dye is able to cross the plasma membrane of the cell only if it is compromised and thus enter the cell nucleus staining it green. However, as Plasmodium schizonts due to their increased membrane permeability, the Syto-16 dyes labels living schizonts as dead by staining their nuclei green. [file 1475-2875-12-190-S2.jpeg]

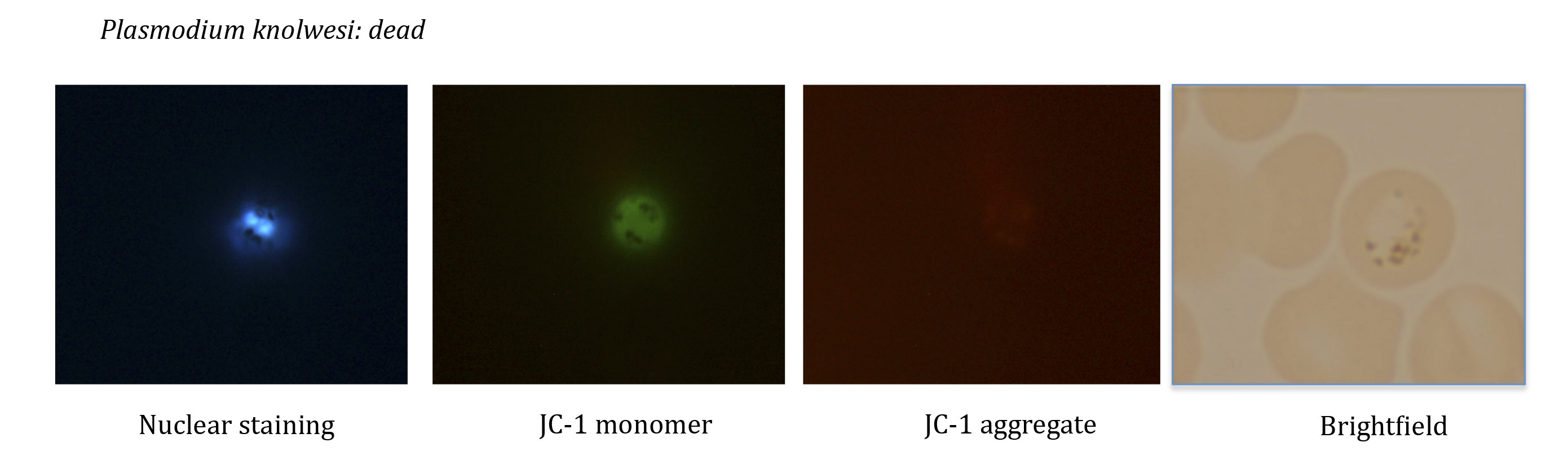

Supplement: Additional file 3 — Dead P. knowlesi parasites stained with JC-1. JC-1 staining of drug-treated Plasmodium knowlesi culture showing a dead parasite as can be appreciated by the absence of signal at 568 nm. [file 1475-2875-12-190-S3.jpeg]

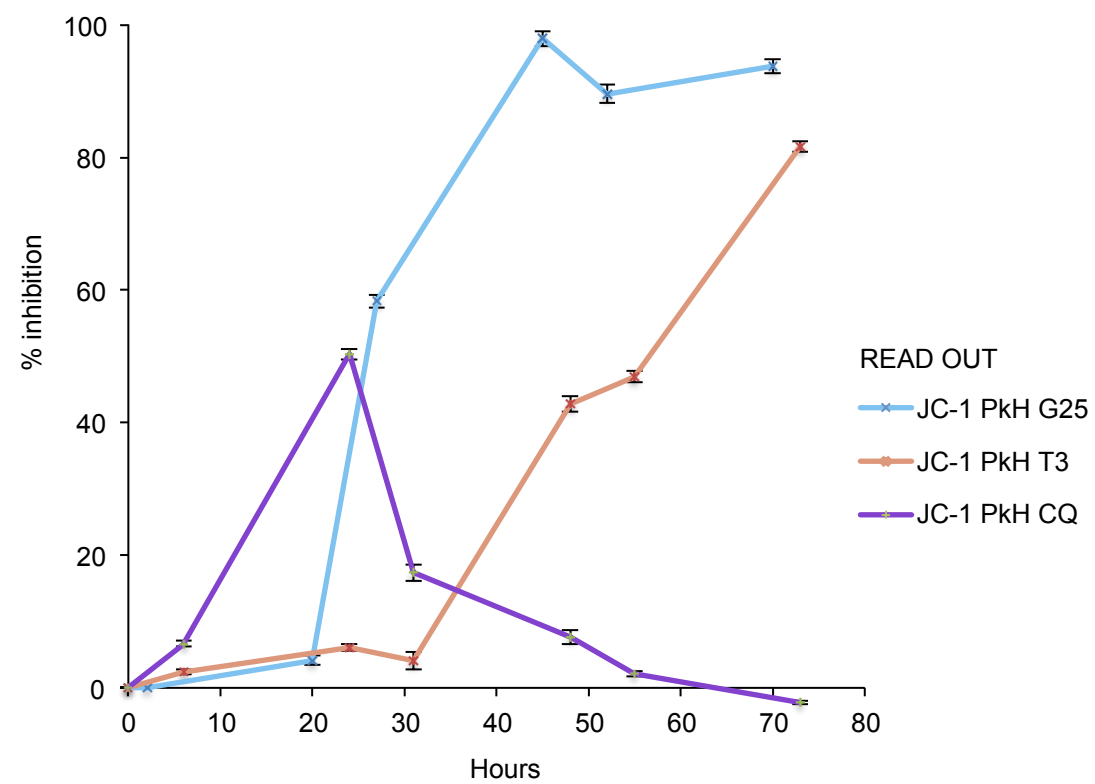

Supplement: Additional file 4 — Delayed death phenotypes monitored using JC-1 on synchronized P. knowelsi H cultures exposed to chloroquine and the phospholipid pathway inhibitors prototype drug.P. knowlesi H culture (5% hematocrit and 0.1 starting parasitaemia) were exposed to 5.62 nM chloroquine and the phospholipid pathway inhibitors prototype drugs (1.38 nM albitiazolium and 0.36 nM G25). After two hours of contact with the drugs, cell were washed and resuspended in drug-free fresh complete medium. JC-1 staining was then used to monitor parasite viability over the course of at least 3 cycles to detect any delayed drug effects. The results are expressed as means ± SEM (n = 3). Exposure of a ring culture to the IC50 of chloroquine for 2 h has no delayed effect but acts on trophozoites and schizonts during the first cycle as expected (violet line), but exposure of the same ring culture to the IC50 of G25 and albitiazolium for only 2 hours results in a potent delayed death effect, which for G25 reaches 100% within the second parasite cycle (blue line) and for albitiazolium probably within beyond the third (red line) cycle. [file 1475-2875-12-190-S4.pdf]
